# Daily iron supplementation does not impact on prevalence of exclusive breastfeeding or growth in young breastfed Gambian infants

**DOI:** 10.1136/bmjnph-2023-000847

**Published:** 2025-01-11

**Authors:** Isabella Stelle, Mamadou Bah, Hans Verhoef, Sophie Moore, Carla Cerami

**Affiliations:** 1 Department of Women and Children's Health, King's College London, London, MO, UK; 2 MRC Unit, The Gambia at LSHTM, Banjul, Banjul, Gambia; 3 Wageningen University, Wageningen, the Netherlands; 4 Women and Children's Health, King's College London, London, UK; 5 Nutrition and Planetary Health, MRC Unit The Gambia at LSHTM, Banjul, Fajara, Gambia

**Keywords:** Nutritional treatment, Malnutrition

## Abstract

**Background:**

In a randomised placebo-controlled trial among exclusively breastfed rural Gambian infants aged 6–10 weeks at randomisation, daily iron supplementation for 14 weeks improved iron status. This secondary analysis explores the impact of iron supplementation on duration of exclusive breastfeeding and growth.

**Methods:**

Breastfed 6–10 week-old infants were supplemented for 14 weeks with either daily iron or placebo (n=101). Infant feeding practices were assessed weekly through questionnaires. Survival analysis was used to measure the effect of iron supplementation on age at and time to cessation of exclusive breastfeeding. Groups were also compared regarding the change in anthropometric z-scores between baseline and endline.

**Results:**

At endline, 31% (n=31/101) of infants were exclusively breastfed. There was no evidence that iron supplementation reduced the time to cessation of exclusive breastfeeding (median: 70 days (range: 7–105 days), iron: 67 days; placebo 71 days; Kaplan-Meier, log-rank test: p=0.15; Cox regression, crude HR: 1.42, 95% CI: 0.86 to 2.34, p=0.17; HR adjusting for infant age and sex: 1.40, 95% CI: 0.85 to 2.31, p=0.19) or age at cessation of exclusive breastfeeding (median time: 18 weeks (range:1–24 weeks), iron: 16 weeks; placebo 18 weeks; Kaplan-Meier, log-rank test: p=0.13; crude HR=1.47, 95% CI: 0.89, 2.43; p=0.13; HR adjusting for infant age and sex=1.44, 95% CI: 0.87, 2.39 p=0.16) There was no evidence that iron supplementation affected infant weight (p=0.79) or length (p=0.64) at endline or change in z-scores during the intervention period for weight-for-age (p=0.99), length-for-age (p=0.70) and weight-for-length (p=0.89). There was no evidence that duration of exclusive breastfeeding impacted endline anthropometric outcomes.

**Conclusion:**

Although requiring replication in larger trials, these findings do not raise concerns about iron supplementations’ effect on feeding or growth in exclusively breastfed infants.

**WHAT IS ALREADY KNOWN ON THIS TOPIC**

**WHAT THIS STUDY ADDS**

- This secondary analysis of a randomised placebo-controlled trial among exclusively breastfed rural Gambian infants aged 6–10 weeks at randomisation, supplemented daily with iron or placebo for 14 weeks, suggests that giving iron to exclusively breastfed infants does not adversely impact on infant feeding patterns or growth.

**HOW THIS STUDY MIGHT AFFECT RESEARCH, PRACTICE OR POLICY**

- These findings add to the few studies around iron supplementation, feeding, and growth in this early age group.

##  Introduction

A recent trial (‘Iron Babies’) in rural Gambia comparing daily supplements of 7.5 mg/day iron as ferrous sulphate or placebo as sorbitol solution USP 70% for a duration of 98 days starting at 6–10 weeks of age was conducted.^1^ Infants were supplemented instead of mothers, due to the already routine antenatal iron folic acid supplementation in The Gambia. The primary objective was to measure the impact of daily iron supplements on serum iron concentration at the end of the intervention. This trial showed that supplementary iron led to an increased serum iron concentration (crude difference in means: 2.5 µmol/L; 95%CI: 0.6 to 4.3 µmol/L, p=0.009) and meaningfully improved additional markers of iron and haematological status, with no evidence of differences in adverse events or deaths reported between trial arms.^1^ This was the first time such an intervention was undertaken in this age group for this population and adds to the very limited number of previously published trials of iron supplementation in exclusively breastfed infants particularly in low-income settings.^2^

The previously highlighted *Iron Babies* trial main results are published elsewhere.^1^ This present study presents a secondary analysis of this trial, exploring the impact of iron supplementation on duration of exclusive breastfeeding and growth.

The WHO recommends exclusive breastfeeding until 6 months of age, and continued breastfeeding until 2 years of age or beyond.^3^ In The Gambia, the prevalence of exclusive breastfeeding at 6 months of age has previously been reported at 32%.^4^ In a nationally representative survey, over half of Gambian infants under 6 months of age (54%) were exclusively breastfed and those living in rural areas were breastfed for a longer duration.^5^ These high rates of exclusive breastfeeding until 6 months of age are largely thanks to the community care work of the National Nutrition Agency in The Gambia. There is concern that mothers may be confused by seemingly contradictory messages that (a) they should administer oral iron supplements; (b) they should not administer any foods or liquids (to adhere to exclusive breastfeeding).

Parallel to possible effects of iron on exclusive breastfeeding, more rapid growth has been correlated with a faster decline in infant iron status,^6^,^9^ although evidence around the impacts of supplementation on growth is unclear. Additionally, studies in infants under 6 months of age are lacking.^2^ Studies in infant populations over 6 months of age with a high prevalence of iron deficiency anaemia have shown a beneficial impact of iron on growth.^10^,^12^ Other studies found no effect, with some even reporting adverse effects.^1113^,^15^ However, these impacts were only seen in iron replete children.^14 15^ A 2020 systematic review of multiple micronutrients and/or iron supplementation in infants under 6 months of age concluded that infants less than 6 months of age benefit biochemically from early supplementation with iron, but the impacts on growth, morbidity and/or mortality and neurobehavioural outcomes remain unclear.^2^ However, this review was limited by the small number of studies looking at supplementation of infants under 6 months of age.

The present study aims to measure the effect of iron supplementation on (a) time to cessation of exclusive breastfeeding; (b) age at cessation of exclusive breastfeeding; (c) linear and ponderal growth; as well as examining the impact of age at baseline and sex on anthropometry. We expected that daily iron supplementation for 98 days would not adversely impact on infant feeding practices or growth in breastfed Gambian infants.

## Methods

Full details of the *Iron Babies* trial can be found in the published trial protocol^16^ with additional details on the trial results in the manuscript on the primary trial outcome.^1^ A summary of the key methods for this analysis is provided below.

### Study setting

Data collection for this study took place in Jarra West in the Lower River Region of The Gambia, West Africa, and was conducted by the Medical Research Council Unit The Gambia at the London School of Hygiene and Tropical Medicine (MRCG@LSHTM).

### Study design and field implementation

Participants were identified at vaccination clinics as well as in the communities in Jarra West by the field staff. After written informed consent was obtained, infants were screened for eligibility at the Jarra Soma Health Centre. Inclusion criteria were healthy infants aged between 6 and 10 weeks, being exclusively breastfed with mothers reporting that they planned to continue breastfeeding through to 6 months of age. Birth weight, collected to the closest 100 g, and gestational age at birth were recorded from birth records. Low birth weight infants (less than 2.5 kg) or infants born prematurely (less than 37 weeks) were included. Further inclusion and exclusion criteria can be found in the published trial protocol.^16^

Infants were individually and randomly allocated on a 1:1 ratio to daily oral supplementation for 98 days with either iron or placebo. Supplements were administered as drops from a liquid formulation containing either iron (7.5 mg as ferrous sulphate contained in 0.5 mL of a sorbitol solution USP 70%,) or its placebo (0.5 mL of sorbitol solution USP 70%). Infants were visited daily for supplement administration in their homes for the duration of the trial by field assistants.

Daily questionnaires assessing infant health were administered throughout the study duration by field staff to mothers. Weekly questionnaires were administered to collect data on breastfeeding practices. Exclusive breastfeeding was defined as per WHO recommendations as no substance outside of breastmilk, besides drops or syrups containing vitamins, minerals, supplements, or medicine, given during the infants first 6 months of life.^17^ Data on adverse events were collected up to 2 weeks post-supplementation. Infant length and weight were measured at baseline (day 1) and endline (day 99). Recumbent length was measured using a Seca 417 length board to the nearest 0.1 cm. Weight was measured using a Seca 336 digital weighing scale to the nearest 10 g, with the infant in minimal clothing.

### Data processing and analysis

Data were downloaded from the REDCap database into Microsoft Excel, where they were collated, cleaned and prepared for analysis. The process of cleaning involved removal of any unrealistic outliers (such as unfeasible anthropometric measurements). At each weekly survey visit, infants were coded as exclusively breastfed or not. Statistical analyses were performed using STATA (V.17).

Study groups were described using conventional summary statistics (eg, means or counts with SD). Z-scores for weight-for-age (WAZ), length-for-age (LAZ), weight-for-length (WLZ) were generated to indicate how far a child’s measurement deviates from the median value of the WHO reference population of breastfed infants with the same age and sex.^18^ This was done for growth data collected both at baseline and endline, using the STATA ‘zscore06’ command and cross-checked using WHO Anthro software.^19^

Kaplan-Meier analysis (with log-rank tests) and Cox regression analysis were used to measure the effect of intervention (ie, iron supplementation) on the time from randomisation until cessation of exclusive breastfeeding, defined as the first-recorded instance of introduction of a non-breastmilk feed. For infants who did not experience this outcome (ie, they were exclusively breastfed by the end of the intervention period) or who were lost to follow-up, observation time was censored at the time at which they were last observed. A secondary survival analysis was used to measure the effect of intervention on age of cessation of exclusive breastfeeding, defined as age in weeks of the first recorded instance of introduction of a non-breastmilk feed.

Linear regression analysis was used to compare groups regarding the change in z-scores, and analysis of variance to measure group differences in the proportion of infants being underweight (WAZ <−2 SD), stunted (LAZ <−2 SD) and wasted (WLZ <−2 SD) at endline.^20^ As well as explore the impacts of sex and age at baseline, and mode of feeding and length of time of exclusive breastfeeding, (adjusted for baseline variables selected a priori: infant sex and age) on infant growth outcomes.

## Results

**Figure 1:**
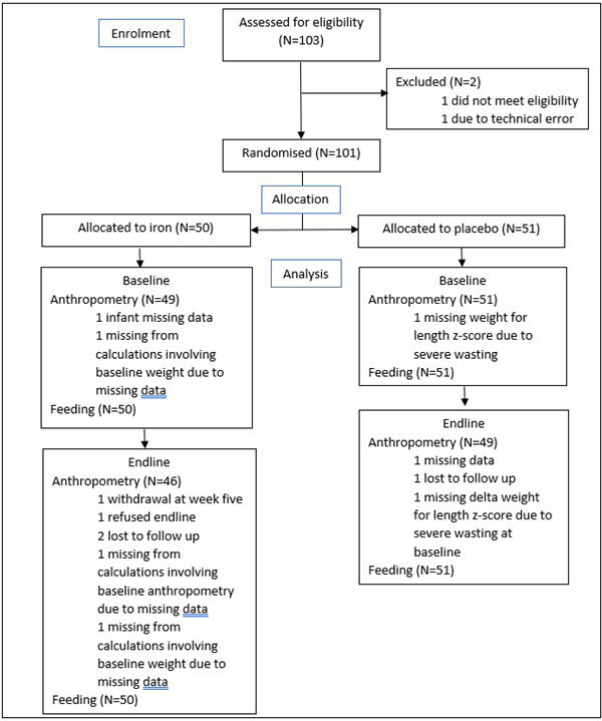
CONSORT diagram. CONSORT, Consolidated Standards of Reporting Trials.

Of 103 infants who were screened for eligibility, 101 were randomised to receive either iron or placebo. Participant flow through the trial is described in figure 1. Because few data were missing, we used complete-case analysis to handle incomplete data. This procedure produces unbiased estimates if data are missing completely at random. For seven infants, data on breastfeeding status were missing at endline; of those, five infants also had missing anthropometry indices missing at endline.

Baseline infant and maternal characteristics are summarised in table 1. On average, infants were aged 7.5 weeks (range: 6.1–10.0 weeks); the prevalence of being underweight, stunted and wasted was 12.1%, 9.0% and 5.1%, respectively. Mean maternal age was 26.4 years and median parity was 4 (IQR: 2.5) children. A little over half (52%) of the mothers had not completed primary school. The mean (SD) length of enrolment was 99.0 (2.45) days (range: 78.0–105 days, due to late collection of endline data). One infant moved away from the study area after 78 days of enrolment, so the infant’s endline data were collected after 78 days. Study groups were similar regarding birth weight (mean (SD): 2.99 (0.51) vs 2.97 (0.64) kg), baseline age (7.5 (1.0) vs 7.6 (1.1) weeks), weight (4.73 (0.72) vs 4.75 (0.81 kg) and length (55.8 (2.7) vs 55.7 (3.6) cm) between the iron and placebo groups.

**Table 1:** Baseline characteristics for infants and their mothers, by study group

	Entire cohort	Iron group	Placebo group
**n**	101	50	51
Sex			
Male	45.5% (46)	48.0% (24)	43.1% (22)
Female	54.5% (55)	52.0% (26)	56.9% (29)
Tribe			
Mandinka	73% (74)	74% (37)	73% (37)
Fula	23% (23)	20% (10)	25% (13)
Wollof	3.0% (3)	4.0% (2)	2.0% (1)
Serer	1.0% (1)	2.0% (1)	0.0% (0)
Birth weight (in kg) (SD)	2.97 (0.58) (79)	2.99 (0.51) (36)	2.97 (0.64) (43)
Infant age (in weeks) (SD)	7.5 (1.1) (101)	7.5 (1.0) (50)	7.6 (1.1) (51)
Infant weight (in kg) (SD)	4.74 (0.76) (99)	4.73 (0.72) (48)	4.75 (0.81) (51)
Infant length (in cm) (SD)	55.7 (3.2) (100)	55.8 (2.7) (49)	55.7 (3.6) (51)
Weight for age z-score (WAZ) (SD)	−0.60 (1.18) (99)	−0.59 (1.11) (48)	−0.61 (1.25) (51)
Length for age z-score (LAZ) (SD)	−0.53 (1.38) (100)	−0.52 (1.13) (49)	−0.54 (1.60) (51)
Weight for length z-score (WLZ) (SD)	−0.09 (1.12) (98)	−0.07 (1.14) (48)	−0.11 (1.11) (50)
Underweight^*^	12.1% (12/99)	8.3% (4/48)	15.7% (8/51)
Stunted^*^	9.0% (9/100)	6.1% (3/49)	11.8% (6/51)
Wasted^*^	5.1% (5/98)	2.1% (1/48)	8.0% (4/50)
Maternal age (years)	26.4 (6.1)	24.8 (5.5)	28.0 (6.2)
Maternal parity**†**	4 (2, 5)	4 (2, 5)	4 (2, 5)
Maternal educational level completed			
Less than primary	52% (53)	48% (24)	57% (29)
Primary	16% (16)	16% (8)	16% (8)
Secondary	27% (27)	30% (15)	23% (12)
Higher	5.0% (5)	6.0% (3)	4.0% (2)

Values indicate percentage (n) or mean (SD) (n).

* WHO definition of underweight (WAZ <−2 SD), stunting (LAZ <−2 SD) and wasting (WLZ <−2 SD).^20^

† Maternal parity is expressed as median (IQR range). Z-scores were calculated using WHO Anthro.^19^

### Infant feeding practices

Of the 101 infants who were randomised, 31% (n=31) were still being exclusively breastfed at endline (n=13 iron, n=18 placebo), while 62% (n=63) were partially breastfed (n=36 iron, n=27 placebo) and 7% (n=7) infants were missing endline data (n=1 iron, n=6 placebo). There was no statistically significant difference in rates of exclusive breastfeeding at endline by treatment group (iron: 26.5%, placebo: 35.4%; p=0.17).

Of 63 infants for whom non-breastmilk feeds were introduced throughout the study, 21 were only given water on top of breastmilk. From the weekly feeding questionnaires across the 14 weeks study period, 535 instances were reported when non-breastmilk feeds were given. When non-breastmilk feeds were given, water was the most common (76% of instances), followed by local foods (7%), powdered milk (6%), local drinks and commercial formula (4% each), powdered potato (2%) and other substances (1%).

**Figure 2:**
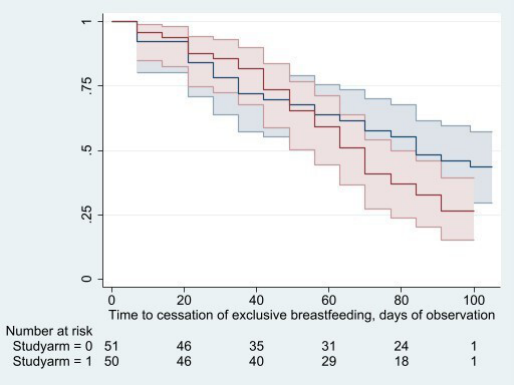
Kaplan Meier curves with 95% CIs and corresponding risk table, by intervention arm (red: iron; blue placebo).

**Figure 3:**
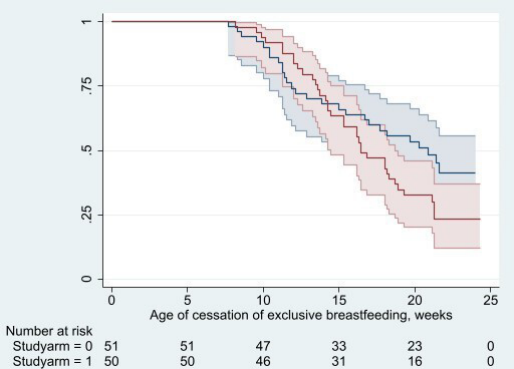
Kaplan Meier curves with 95% CIs and corresponding risk table, by intervention arm (red: iron; blue placebo).

There was no evidence that iron supplementation reduced the time to cessation of exclusive breastfeeding (median: 70 days (range: 7–105 days), iron: 67 days; placebo 71 days; Kaplan-Meier, log-rank test: p=0.15; Cox regression, crude HR: 1.42, 95% CI: 0.86 to 2.34, p=0.17; HR adjusting for infant age and sex: 1.40, 95% CI: 0.85 to 2.31, p=0.19) (figure 2) or age at cessation of exclusive breastfeeding (median time: 18 weeks (range:1–24 weeks), iron: 16 weeks; placebo 18 weeks; Kaplan-Meier, log-rank test: p=0.13; crude HR=1.47, 95% CI: 0.89 to 2.43; p=0.13; HR adjusting for infant age and sex=1.44, 95% CI: 0.87 to 2.39 p=0.16) (figure 3). On average, 9.5 infants stopped exclusive breastfeeding per 1000 infant-days of observation time (iron: 8.0; placebo: 11.2), and 3.7 infants (iron: 4.3; placebo: 3.1) stopped exclusive breastfeeding per 1000 infant-days.

### Anthropometry

When looking at the whole cohort, mean weight and length (SD) at endline were 6.81 (0.95) kg and 63.8 (2.9) cm, respectively. There was a decrease in the number of infants who were underweight and stunted between baseline and endline (underweight: 12.1%, n=12/99 vs 9.47%, n=9/95; stunted: 9.00%, n=9/100 vs 8.42%, n=8/95; for baseline vs endline, respectively) and a marginal increase in the number of infants who were wasted (5.10%, n=5/98 vs 5.26%, n=5/95; for baseline vs endline, respectively).

There was no evidence of meaningful differences between iron and placebo groups in any of the anthropometric outcomes (table 2).

**Table 2:** Crude effect of iron supplementation on anthropometric outcomes

	Entire cohort	Iron group	Placebo group	P value: iron vs placebo	Group difference (95% CI)
Infant age (in weeks) (SD)*	21.7 (1.1) (100)	21.7 (1.0) (49)	21.7 (1.2) (51)	–	0.18 (−3.03 to 3.38)
Weight (in kg) (SD)*	6.81 (0.95) (95)	6.83 (0.97) (46)	6.78 (0.95) (49)	0.79	−0.05 (−0.44 to 0.34)
Length (in cm) (SD)*	63.8 (2.9) (95)	63.6 (2.8) (46)	63.9 (3.0) (49)	0.64	0.28 (−0.89 to 1.45)
Weight for age z-score (WAZ) (SD)*	−0.52 (1.19) (95)	−0.49 (1.26) (46)	−0.54 (1.12) (49)	0.83	−0.05 (−0.54 to 0.43)
Length for age z-score (LAZ) (SD)*	−0.50 (1.18) (95)	−0.57 (1.21) (46)	−0.43 (1.16) (49)	0.57	0.14 (−0.35 to 0.62)
Weight for length z-score (WLZ) (SD)*	−0.16 (1.08) (95)	−0.08 (1.09) (46)	−0.25 (1.07) (49)	0.44	−0.17 (−0.61 to 0.27)
Change in WAZ (SD)*	0.11 (0.76) (93)	0.11 (0.79) (44)	0.11 (0.74) (49)	0.99	0.00 (−0.31 to 0.32)
Change in LAZ (SD)*	0.09 (1.05) (94)	0.05 (1.01) (45)	0.13 (1.08) (49)	0.70	0.08 (−0.35 to 0.51)
Change in WLZ (SD)*	−0.10 (1.31) (92)	−0.08 (1.39) (44)	−0.12 (1.24) (48)	0.89	−0.04 (−0.58 to 0.51)
Underweight††	9.47% (9/95)	8.70% (4/46)	10.2% (5/49)	0.80	0.02 (−0.11 to 0.14)
Stunted††	8.42% (8/95)	8.70% (4/46)	8.16% (4/49)	0.93	−0.01 (−0.12 to 0.11)
Wasted††	5.26% (5/95)	6.52% (3/46)	4.08% (2/49)	0.60	−0.02 (−0.12 to 0.07)

Z-scores were calculated using WHO Anthro.^19^

* Expressed as mean (SD) (n).

† WHO definition of underweight (WAZ <−2), stunting (LAZ <−2) and wasting (WLZ <−2).^24^

When looking at the impact of infant sex and age at baseline on growth, there was statistical evidence for an association between infant sex and change in WAZ, with males showing a greater decrease in WAZ over the duration of the trial than females (p=0.03, coefficient: −0.34, 95% CI: −0.65 to –0.03). The mean (SD) change in WAZ for males was −0.08 (0.70), verses 0.26 (0.78) for females. Males’ mean (SD) WAZ changed from −0.46 (0.87) at baseline to −0.55 (1.03) at endline, whereas females changed from −0.70 (1.38) to −0.49 (1.31). A statistical association was observed between infant age at baseline and change in LAZ with younger infants at baseline having greater change in LAZ (p=0.01, coefficient: −0.26, 95% CI: −0.45 to –0.06) over the duration of the trial than older infants. No other associations with z-scores and infant age or sex at baseline were observed. There was no statistical evidence for associations between intervention arm and endline anthropometry outcomes for both unadjusted and adjusted (sex and age at baseline) models.

There was no statistical support in the unadjusted or adjusted model (infant sex and age at baseline), that duration of exclusive breastfeeding had any impact on endline anthropometric outcomes.

## Discussion

This secondary analysis of data collected from a pilot trial in rural Gambia has shown that daily iron supplementation to young, breastfed infants did not adversely impact on patterns of infant feeding or growth. This finding, among a population of breastfed young infants with marginal iron status supports that supplementary iron, does not impact on mode of feeding, does not compromise infant growth, and adds to our previously published observations that infant iron status is significantly improved in this age group.^1^

The prevalence of exclusive breastfeeding in this setting at 6 months of age has previously been reported at a similar rate as found in this trial;^4^ however, when looking at country wide statistics, the rates of exclusive breastfeeding in The Gambia are reported as higher than in this trial.^5^ All infants were exclusively breastfeeding at entry into the trial and only 31 remained exclusively breastfeed until the end of the trial, 63 remained partially breastfed, and seven had unknown endline feeding status. These lower than the nationally average rates of exclusive breastfeeding until 6 months of age in the *Iron Babies* cohort could be due to the participants proximity to a large town—Soma.^5^ Rates of exclusive breastfeeding have been reported to be higher (closer to 50%) in more rural regions of The Gambia.^5^ In the *Iron Babies* cohort, instances when non-breastmilk feeds were given, the most common substance was water, 76% of the time. Formula had a low per cent of usage (4% of instances), whereas local foods (7%) or powdered milk (6%) were more often given. In a neighbouring rural region, watery millet cereals have been noted to be introduced as early as 3 months of age.^21^ In *Iron Babies,* the remaining non-breastmilk feeds consisted of local juices (4%), powdered potato (2%) and other substances (1%). Supplementary iron did not impact any of the infant feeding practices monitored. A systematic review of iron and/or multiple micronutrient interventions in infants under 6 months of age, found no side effects around infant feeding practices.^2^ However, a systemic review of caregiver perspectives of early nutrition interventions in infants under 24 months of age noted increased infant appetite as the main reason for high acceptability of interventions.^22^

Consistent with published data from rural Gambia, infant z-scores decreased with age in this cohort, reflecting growth faltering across infancy.^4 23^ Infant mean birth weight in *Iron Babies* was 2.97 kg, which is similar to previously reported in a cohort of rural Gambian infants.^4^

Previous trials and commentaries have raised concerns that supplementary iron given to young, breastfeeding infants may impact on development or feeding.^2 24^ However, overall data are lacking in this age cohort. There was no statistical support for any growth impacts due to iron intervention in this study. Some studies in infant populations over 6 months of age have reported adverse effects from iron on growth, but these impacts were only seen in iron replete children.^18 19^ In this study, duration of exclusive breastfeeding did not impact endline anthropometric outcomes.

This study has several limitations. The primary limitation is the sample size of this pilot study which was not powered to test the secondary outcomes considered in this paper. Nevertheless, given the concerns around the safety of early iron interventions and the lack of data from well-conducted trials in this age group, ensuring supplemental iron has no apparent negative impacts on feeding practices and infant growth is important. Daily administration of the intervention by the field workers, while ensuring efficacy, may have impacted on caregivers’ usual behaviours, especially in relation to feeding practices. A qualitative adjunct study found that feasibility to scale up with self-administration was a concern for some involved in the study, but overall, there was enthusiasm for the intervention.^24^ In the survival analysis, the 95% CI was about 2.5 on the upper end, so a larger study would be required to verify that intervention did not have an impact on feeding practices. Additionally, in the Kaplan Meier curves, by the end of the analysis time, the iron group sees an earlier time since enrolment of introduction of non-breastmilk feeds as well as earlier age of cessation of exclusive breastfeeding, and while the p values indicate this is due to chance in these graphs, this should be explored in larger studies. Another important limitation worth noting was maternal self-reporting to field staff on infant feeding practices. While not breastfeeding data, self-reported dietary recall data are widely understood to be of limited validity.^25^ Finally, infant anthropometric indices were only assessed at baseline and endline so more sophisticated growth modelling could not be performed.

## Conclusion

In summary, this study has shown that giving daily iron from 6 to 10 weeks of age to breastfed, rural Gambian infants improved several markers of iron status without any adverse impacts on the prevalence of exclusive breastfeeding or added risk to growth.^1^ No adverse effects were seen on growth outcomes from the early iron intervention, adding to the few studies done in the early age group. These findings are important as, together with the primary trial outcomes they add to the evidence of importance of early iron interventions in contexts of marginal status and negate any concerns around the impact of iron supplementation on feeding practices and growth.^1^ Although requiring replication in larger trials, these findings suggest that giving iron to young breastfeeding infants, in addition to having positive effects on iron status, does not adversely impact on infant feeding patterns or growth.

## Data Availability

Data are available upon reasonable request.
